# Reconstruction and applications of consensus yeast metabolic network based on RNA sequencing

**DOI:** 10.1002/2211-5463.12033

**Published:** 2016-02-27

**Authors:** Yuqi Zhao, Yanjie Wang, Lei Zou, Jingfei Huang

**Affiliations:** ^1^ State Key Laboratory of Genetic Resources and EvolutionKunming Institute of ZoologyChinese Academy of SciencesYunnanChina; ^2^ Key Laboratory of Animal Models and Human Disease Mechanisms of Chinese Academy of Sciences and Yunnan ProvinceKunming Institute of ZoologyChinese Academy of SciencesYunnanChina; ^3^ Department of General SurgeryFirst People's Hospital of Yunnan ProvinceKunmingChina; ^4^ Collaborative Innovation Center for Natural Products and Biological Drugs of YunnanKunmingYunnanChina

**Keywords:** metabolic engineering, metabolic network, RNA sequencing, *Saccharomyces* species

## Abstract

One practical application of genome‐scale metabolic reconstructions is to interrogate multispecies relationships. Here, we report a consensus metabolic model in four yeast species (*Saccharomyces cerevisiae*,* S. paradoxus*,* S. mikatae*, and *S. bayanus*) by integrating metabolic network simulations with RNA sequencing (RNA‐seq) datasets. We generated high‐resolution transcriptome maps of four yeast species through *de novo* assembly and genome‐guided approaches. The transcriptomes were annotated and applied to build the consensus metabolic network, which was verified using independent RNA‐seq experiments. The expression profiles reveal that the genes involved in amino acid and lipid metabolism are highly coexpressed. The diverse phenotypic characteristics, such as cellular growth and gene deletions, can be simulated using the metabolic model. We also explored the applications of the consensus model in metabolic engineering using yeast‐specific reactions and biofuel production as examples. Similar strategies will benefit communities studying genome‐scale metabolic networks of other organisms.

**Abbreviations**
DEG: Differentially Expressed Genes
FBA: Flux Balance Analysis
GDLS: Genetic Design through Local Search
lMOMA: Linear Minimization Of Metabolic Adjustment
MOMA: Minimization Of Metabolic Adjustment
PCC: Pearson's correlation coefficient
PYRDC: Pyruvate decarboxylase
RPKM: Reads Per Kilobase of exon per Million reads

The metabolic network reconstructions can be applied in diverse aspects, such as metabolic engineering for biotechnological productions 1, and interrogation of multispecies relationships 2. Among these applications, the cross‐species comparison of metabolic networks allow for a systematic investigation of structure‐function relationships of biological processes and may put forth valuable hints on the evolution of these metabolic pathways 3, 4, 5. For instance, the consequences of copy number alterations in metabolic networks suggested a potential role for dosage selection in the mammalian evolution 3. In addition, a ‘community consensus’ reconstruction of the yeast metabolic network was performed by Herrgård *et al*. 6, which was based on a large, focused work meeting, to define the protocol for the curation process as well as resolving the majority of discrepancies between the existing reconstructions.

However, the majority of the current metabolic networks are reconstructed based on the prior knowledge (knowledge‐driven) and focused on network topologies or characteristics of components in the model, ignoring the actual states of the metabolic networks 7. For instance, the transcriptomes in cells should always be considered, in which the changes are critical for generating phenotypic diversity among species 8. On the other hand, the gene expression divergence may lead to the failures of repeating biological experiments or of developing medicines in scientific research field 8. Recent advances in sequencing technology have made it available to derive accurate metabolic models 9, 10. In theory, RNA‐seq can be applied to reconstruct complete and high‐resolution transcriptomes across all species, cell types and states 11, 12, 13. Several methods have been developed to build the transcriptome, and they fall into two main classes: ‘genome‐guided’ and genome‐independent (*de novo* assembly) 14. The first methods rely on a reference genome to first map all the RNA‐seq reads to the genome and then assemble overlapping reads into transcripts. Unfortunately, the genome‐guided method does not always work. Despite the large drop in the cost of next‐generation sequencing, the complete study of a genome is still costly and difficult, especially for nonmodel organisms. Besides, the model being studied is sufficiently different from the reference genome because it comes from a different strain or line. In this situation, *de novo* assembly is suitable for the accurate reconstructions.

In the study, we carried out an integration analysis of RNA‐seq expression profiles from four yeast species (*Saccharomyces cerevisiae*,* Saccharomyces paradoxus*,* Saccharomyces mikatae*, and *Saccharomyces bayanus*) to generate a ‘consensus’ metabolic network. First, we generated high‐resolution transcriptome maps of four yeast species through *de novo* assembly and genome‐guided approach. We then produced a consensus metabolic network and validated the model using an independent RNA‐seq study. By quantifying the gene expression level in the model, we estimated the conservation and divergence of metabolic pathways. We also discussed the practical applications of the metabolic model.

## Materials and methods

### Overview

**Figure 1:**
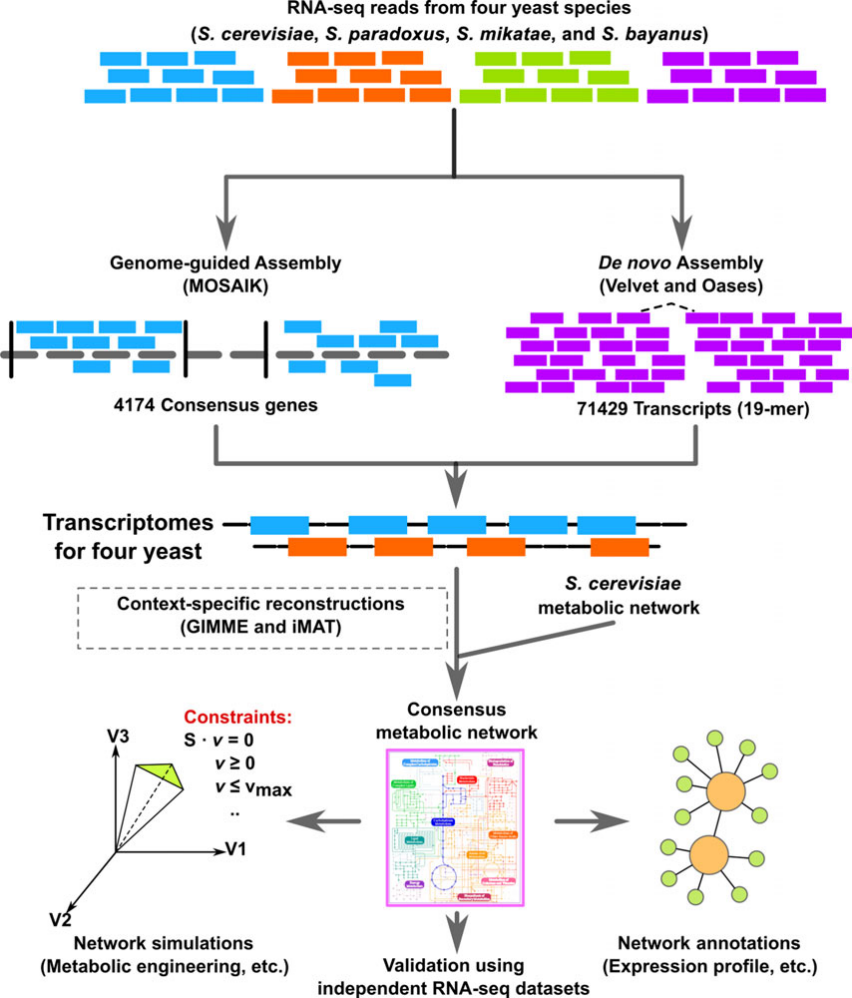
A flowchart schematic representation of our study. The process consists of four steps: (1) genome‐guided transcriptome reconstruction; (2) *de novo* transcriptome assembly; (3) reconstruction of consensus metabolic model; and (4) model validation, simulation, and annotation. In step (4), we determined the conservation and divergence of metabolic genes in gene expression.

The process, explained in detail below and illustrated in Fig. 1, consists of four steps: (a) genome‐guided transcriptome reconstruction; (b) *de novo* transcriptome assembly; (c) reconstruction of consensus metabolic model; and (d) model annotations, simulations, and validation.

### RNA‐Seq data acquisition and metabolic network of *Saccharomyces cerevisiae*

The raw RNA‐seq data from four yeast species (*S. cerevisiae*,* S. paradoxus*,* S. mikatae*, and *S. bayanus*) was downloaded from the NCBI GEO database (NCBI GEO accession: GSE32679) 15, with two replicates for each species. The four yeast species were grown in complete media and sampled according to the protocol described in Marth *et al*.'s study 15.

The *S. cerevisiae* iND750 metabolic network (hereinafter referred to as ‘iND750′) was retrieved from Schellenberger *et al*.'s work 16. It contains 1061 metabolites, which participate in 1266 reactions in 47 subsystems, catalyzed by 750 verified *S. cerevisiae* genes. The model is available as a systems biology markup language (SBML) 17 file, which could be easily used in the MATLAB‐compatible COBRA Toolbox 18.

### Genome‐guided transcriptome reconstruction

The reference genome and annotations for *S. cerevisiae* were obtained from the Saccharomyces Genome Database (http://www.yeastgenome.org/). Genome, annotations, and orthology mappings for the other species were from Kellis *et al*.'s work 19. RNA‐seq reads were aligned to their respective genomes using the mosaik alignment program (version 1.1.0018, http://bioinformatics.bc.edu/marthlab/Mosaik) allowing for a threshold of two mismatches between each 35‐base pair read and the reference genome. The alignment output was parsed using the bamtools API 20.

### *De novo* transcriptome assembly

All the *de novo* assemblies were run with oases (version 0.2.08) 21, and velvet (version 1.2.07) 22. In each of the assemblies, the RNA‐seq reads were used to build a de Bruijn graph through Velvet, with the k‐mer lengths of 17, 19, 21, 23, 25, 27, 29, 31, and 33, respectively. The Bruijn graphs were then simplified for errors, organized into a scaffold, divided into loci, and finally analyzed to extract transcript assemblies or transfrags. Once all of the individual k‐mer assemblies were finished, they were merged into a final assembly.

### Reconstruction of consensus metabolic model

In the process, the blastn (version 2.2.23) program was used to map all the metabolic genes in *S. cerevisiae* model to the transcriptomes reconstructed through genome‐guided approach and *de novo* assembly, with e‐value threshold set at 10^−4^. We then overlapped all the positive results from four species to obtain a consensus metabolic gene dataset.

We derived a consensus metabolic model from the *S. cerevisiae* metabolic network using two commonly used algorithms of top–down metabolic reconstructions, including GIMME 7 and iMAT 23. The GIMME algorithm is a linear programming procedure, which can matches high‐throughput omics data (such as transcriptome and proteome) to an original flux distribution that obtained from the full metabolic model. The constraining reactions in the consensus were set up according to the Yeast 5 genome‐scale model 24. On the other hand, the iMAT algorithm is a mixed integer linear programming algorithm that best matches the omics data to pathway length. Using these two methods for the consensus model reconstruction, (a) we prepared the *S. cerevisiae* metabolic network, and (b) the high‐quality transcriptomes in four species based on the genome‐guided and *de novo* methods above.

### Differential expression in pairwise comparison

We identified differentially expressed genes (DEGs) using a free bioconductor 25 package, called deseq (version 1.1.11) 26. In RNA‐seq experiments, read counts differ for each sample due to a variable number of reads produced by sequencing runs and the mixture of RNA within samples. Sample normalization is confounded by differences in gene expression. To solve the problem, DEseq uses a generalization of the Poisson model, the negative binomial distribution, to model biological and technical variance and test for differential expression between the two conditions or species. All genes that were found to be DEGs between two species (at a *P*‐value cutoff of 0.05 and fold‐change < 0.5 or > 2, that is, fold‐change < −1 or > 1 after log transformation) were retained for further analysis. We also applied reads per KB per million reads (RPKM) to detect gene expression levels 27.

To reduce the bias induced by randomization, we introduced the phastCons tree model 28 with branch lengths for all four species (Fig. S1), with *S*. *bayanus* included in the further analysis as an outgroup. If a DEG between *S*. *bayanus* and the other three species show the same tendency (i.e., all the fold changes > 2 or < 0.5), the gene was considered to form divergent expression patterns.

### Validation of the metabolic model using independent RNA‐seq study

To validate our metabolic model, we introduced another independent RNA‐seq dataset (NCBI GEO accession: GSE38875), with two replicates for *S. paradoxus*,* S. mikatae*, and *S. bayanus*, and six replicates for *S. cerevisiae* 29. Cultures were grown at 25 °C in yeast extract peptone dextrose medium to log phase. The RNA‐seq reads were analyzed using the same procedures in Fig. 1: (a) the transcriptomes for the four yeast species were reconstructed using both genome‐guided and *De Novo* assembly methods, and (b) the consensus metabolic model was built using GIMME 7 and iMAT 23 algorithms based on the reconstructed transcriptomes and *S. cerevisiae* iND750 16. Moreover, we compared our metabolic network with manually curated metabolic networks, including YEAST5 30 and YEAST6 31.

### Reconstruction of coexpression network

We combined the two independent RNA‐seq datasets together, and got 20 expression profiles in total for the four yeast species. The genes were ranked according to expression variance among samples and the ones with lowest variance (the top 25 percentile) were filtered. We then adopted the Pearson's correlation coefficient (PCCs) of gene expression patterns to measure the gene coexpression. To make the result solid, we set |Pearson's r| ≥ 0.90.

### Metabolic network simulations

The metabolic models were analyzed by using multiple methods in COBRA toolbox (V 2.0.2) 18, including flux balance analysis (FBA) 32, minimization of metabolic adjustment (MOMA) 33, and Genetic Design through Local Search (GDLS) 34.

These methods provide important tools for harnessing the knowledge encoded in the reconstructed metabolic model. FBA predicts metabolic flux distributions at steady state by using linear programming while the method of minimization of metabolic adjustment (MOMA) employs quadratic programming to identify a point in flux space, which is closest to the wild‐type point, compatibly with the gene deletion constraint. FBA and MOMA of the metabolic network were used to calculate the impact of gene deletions on maximum biomass production rate (a proxy for fitness). We set both the upper and lower flux bounds of the reaction(s) involving the deleted gene to zero. The gene‐reaction associations in the model are indicated by logical relationships between metabolic genes and their corresponding reactions. That is, if a single gene participates in multiple reactions, the gene deletion will result in the removal of all associated reactions. On the other hand, if a reaction involves multiple noninteracting genes, it will not be silenced in a single gene deletion. We categorized the simulation results from a single gene deletion into nonlethal and lethal, which correspond to unchanged maximal growth (defined as mutant growing at > 99.9% of the wild‐type growth rate) and reduced maximal growth or no growth separately. We can obtain valuable information by exploring the effect of reducing flux through a single reaction on growth, for example, predicting the haploinsufficient phenotypes in yeasts. Moreover, robustness analysis of the metabolic network tells us how growth rate changes as the flux through a specific reaction of interest varies in magnitude 35.

The GDLS algorithm 34 was used to identify the reactions list to knock out in order to increase *in silico* production of desired metabolites. GDLS is a computational design tool for metabolic engineering, which uses an efficient, low‐complexity local search approach to identify favorable genetic designs from flux balance metabolic models 34. In this study, we set neighborhood size to be 2, maximum number of knockouts to be 5, and the minimum growth rate to be 0.05 mmol gDW^−1^ h^−1^ 36. The model was adjusted as the minimal medium composition to be aerobic and contain a glucose supply (20 mmol gDW^−1^ h^−1^).

### Essential genes in yeast genome

We retrieved the essential genes for yeast growth from *Saccharomyces* Genome Deletion Project (http://www-sequence.stanford.edu/group/yeast_deletion_project), which generates more than 20000 strains with the overall goal of assigning function to the ORFs through phenotypic analysis of the mutants 37. Based on the gene deletion experiments, the single gene deletion results using FBA and lMOMA can be categorized into: True Positives (model simulation predicts growth when inessential genes are deleted), False Negatives (model simulation predicts no growth when inessential genes are deleted), False Positives (model simulation predicts growth when essential genes are deleted), and True Negatives (model simulation predicts no growth when essential genes are deleted).

### Gene ontology and statistical analysis

All the Gene Ontology enrichment analyses were assessed using ClueGO, a Gene Ontology‐based tool as functionally grouped networks 38. KEGG pathway analysis was also performed using ClueGO. Two‐sided hypergeometric test was adopted as the default statistical test. Tukey–Kramer test was performed to determine the statistical significance of the average length of assembled transcripts from different k‐mers. Two‐sample *t*‐test was performed to compare the gene expression levels between metabolic genes.

## Results

### Integrated analysis of genome‐guided and *de novo* assembly

**Figure 2:**
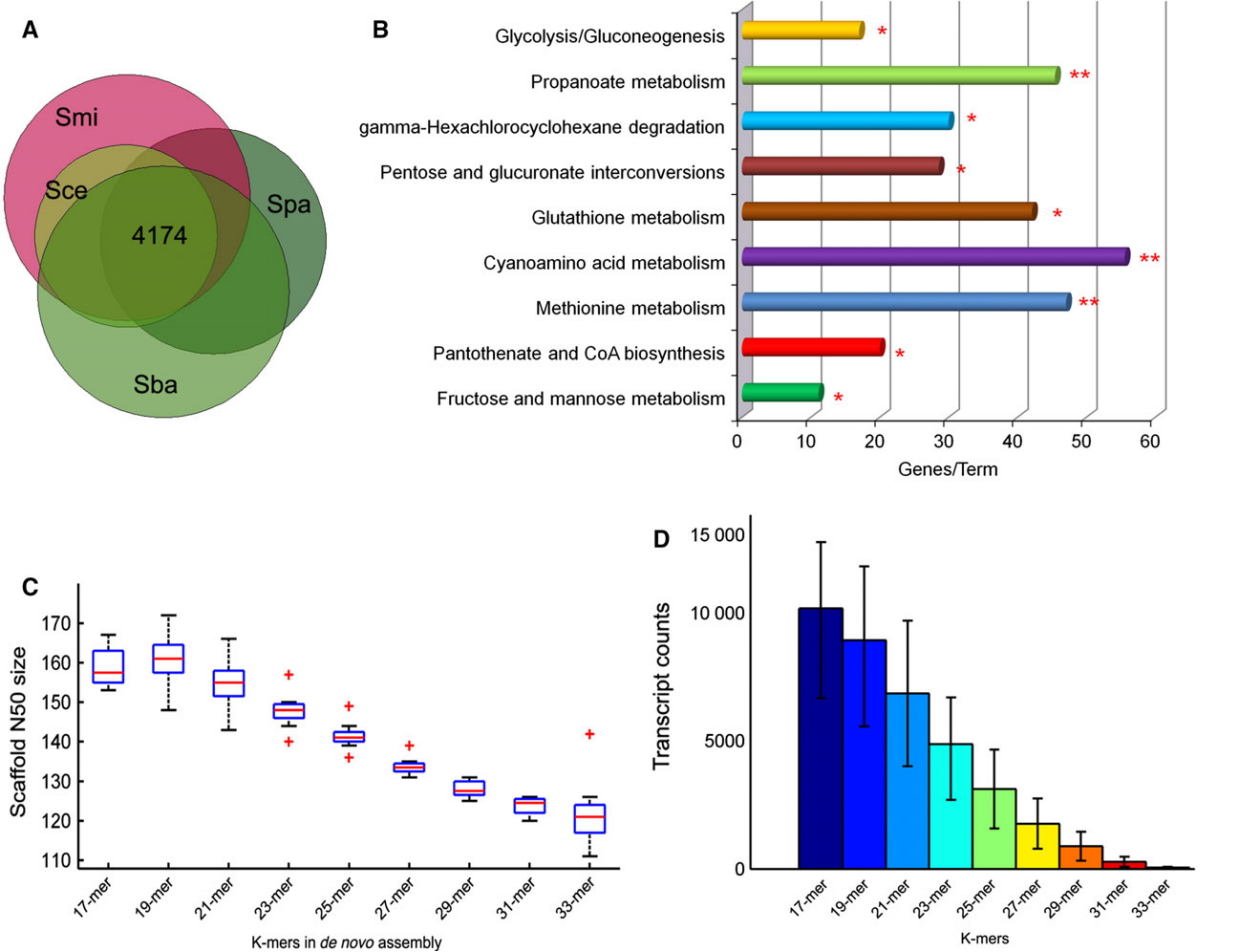
Transcriptome assembly using RNA‐seq reads. (A) Venn plot of transcripts of four yeast species obtained through the genome‐guided method, with 4174 consensus transcripts. Sce, Spa, Smi, and Sba represent *Saccharomyces cerevisiae*,* S. paradoxus*,* S. mikatae*, and *S. bayanus* separately. (B) KEGG pathway analysis of the metabolic genes not included in the consensus enzymes obtained through the genome‐guided method. The red stars indicate statistical significance (two stars when *P* < 0.01; one star when *P* < 0.05). (C) The scaffold N50 values when the k‐mer was set to 17, 19, 21, 23, 25, 27, 29, 31, and 33 in *de novo* assembly. (D) The average transcript count in each species when the k‐mer was set to 17, 19, 21, 23, 25, 27, 29, 31, and 33 in *de novo* assembly.

We searched transcripts of each species obtained through the genome‐guided method against the sequences from the other three species and retrieved 4174 consensus transcripts (Fig. 2A), accounting 78.7% of the annotated cDNA sequences in *S. cerevisiae*. Of these consensus genes, 545 were found to be metabolic genes in *S. cerevisiae* metabolic genes. Meanwhile, molecular function enrichment analysis with Gene Ontology annotation reveals that the transcripts not included in consensus dataset from *S. cerevisiae* are enriched with DNA‐binding (GO:00036770), protein kinase activity (GO:00046720), and transcription factor binding (GO:00081340) and other important function categories (the detailed information was depicted in Fig. S2; all *P* values <0.01), which are crucial for cell growth. KEGG pathway analysis shows that the metabolic genes not included in the consensus dataset were enriched with basic metabolic pathways (Fig. 2B), such as Glutathione metabolism (KEGG:004800), and Glycolysis/Gluconeogenesis (KEGG:000100).

We reasoned that there were missing consensus genes between four yeast species due to the incomplete genome information. As a result, we carried out *de novo* assembly of 2.9 × 10^8^ RNA‐seq reads to generate a high‐resolution transcriptome map. In the single k‐mer assembly in oases and velvet protocol, the average length of scaffolds was highest when the k‐mer was set to 19 (Fig. 2C; Tukey–Kramer test, *P* = 5.7 × 10^−7^). Besides, the transcripts retrieved from 19‐mer performed best in the following BLAST analysis, which were therefore used for the further analysis. Oases assembles 71429 transcripts in four yeast species, with two replicates in each species merged into one transcriptome (Fig. 2D). In these transcripts, we detected 217 consensus metabolic genes included in *S. cerevisiae* model. We combined transcriptomes from genome‐guided and *de novo* assembly strategies and obtained 599 consensus metabolic genes in four yeast species.

### Reconstruction and validation of consensus metabolic network in four yeast species

Based on iND750 and the transcriptomes above, we applied two common methods (Materials and Methods) to reconstruct a consensus metabolic network. It shows that the two methods produced the equivalent network, containing 992 metabolites, which participate in 1104 reactions, catalyzed by 604 metabolic genes (TEXT S1). The model was written in systems biology markup language and could be divided into 47 subsystems (Fig. S3). It is observed that different gene‐protein‐reaction (GPR) relationships are adopted in different subsystems. For example, reactions in oxidative phosphorylation are often catalyzed by multiple genes while genes in fatty acid biosynthesis correspond to multiple reactions.

Using an independent RNA‐seq dataset 29, we repeated the procedure for model reconstruction with all the parameters the same (Materials and Methods). The reconstructed transcriptomes contains 4728 consensus transcripts from genome‐guided assembly approach and 73 661 transcripts from *de novo* assembly approach. A total of 602 consensus metabolic genes were found from the transcriptomes. A metabolic model containing 992 metabolites, 1104 reactions, and 604 metabolic genes was obtained. It shows that the model contains the same GPR relationships as the one in TEXT S1, suggesting that the reconstructed model is robust. Moreover, compared to the manually curated metabolic networks 30, 31, the data‐driven network reconstruction we adopted is stable among different datasets. For example, YEAST6 comprises 1458 metabolites participating in 1888 reactions, which are annotated with 900 yeast genes encoding the catalyzing enzymes 31. Meanwhile, YEAST5 includes 1655 metabolites participating in 2110 reactions 30. Another advantage of the data‐driven strategy is the ease to add additional inputs to the metabolic model. For instance, it would be necessary to consider the actual states (gene expression, DNA methylation, etc.) of the metabolic network.

### Expression patterns of metabolic genes

**Figure 3:**
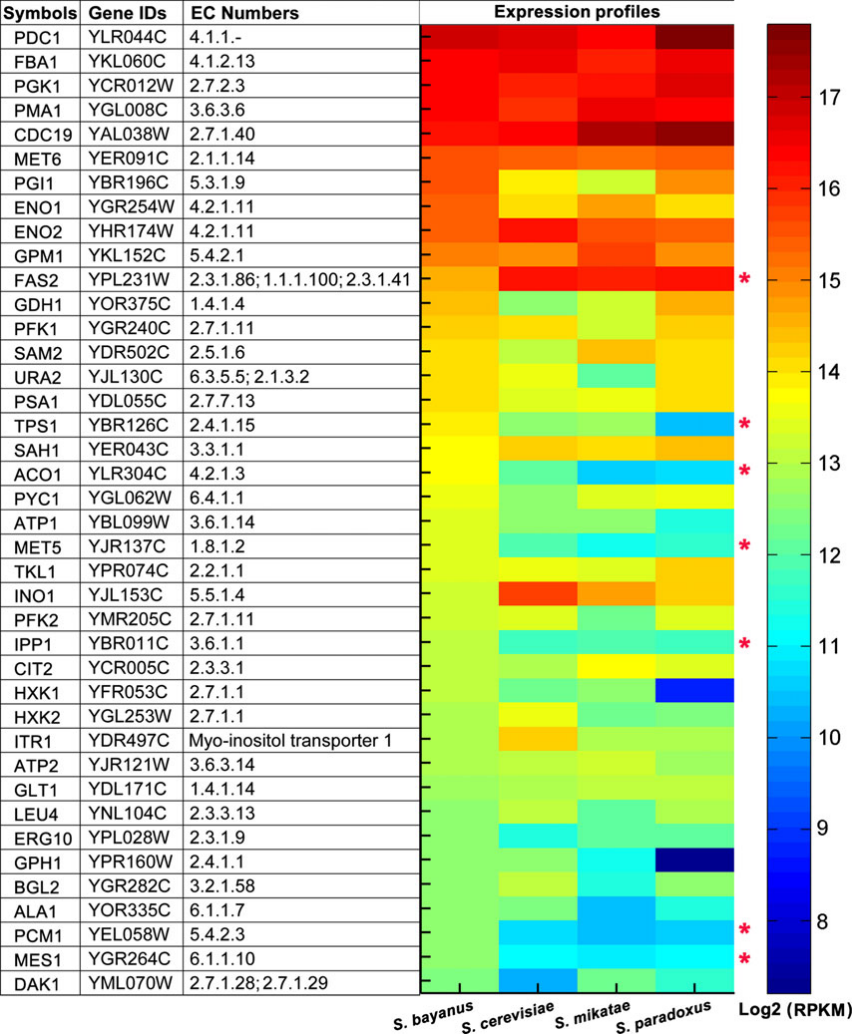
The expression patterns of highly expressed *Saccharomyces bayanus* metabolic genes in four yeast species. The heat map shows the log2 transform of RPKM (reads per kilobase of exon per million reads) values of metabolic genes in the consensus metabolic model. Red stars indicate differentially expressed genes with the changes in RPKM ratio over twofold and *P* < 0.01 between *S. bayanus* and the other three species.

Gene expression is a sensitive measure to observe the molecular change 8. It is commonly accepted that highly expressed genes tend to be essential and evolve at lower rates 39. As a result, we first explored the highly expressed metabolic genes in the yeast consensus metabolic model. Figure 3 shows the top 40 highly expressed metabolic genes in *S. bayanus*, most of which show high expression in other three species. By setting stringent criteria (all fold change ≥ 2 or ≤ 0.5, and *P* < 0.05 between *S. bayanus* and the other three species), we identified seven genes divergent in gene expression. Among these seven genes, gene FAS2 (Fatty acid synthase subunit alpha) shows up‐regulated patterns while the other six genes (ACO1, PCM1, IPP1, MET5, MES1, and TPS1) show down‐regulated patterns between *S. bayanus* and the other three species. It shows that the protein encoded by *S. bayanus* FAS2 is much longer than the orthologous proteins in the other species, with large deletions/insertions in the multiple sequence alignment. We also detected FAS2 with positive selection on coding region (*P* < 10^−5^), using the Nei–Gojobori method 40 and the bootstrap method (500 replicates) in MEGA5 41.

**Figure 4:**
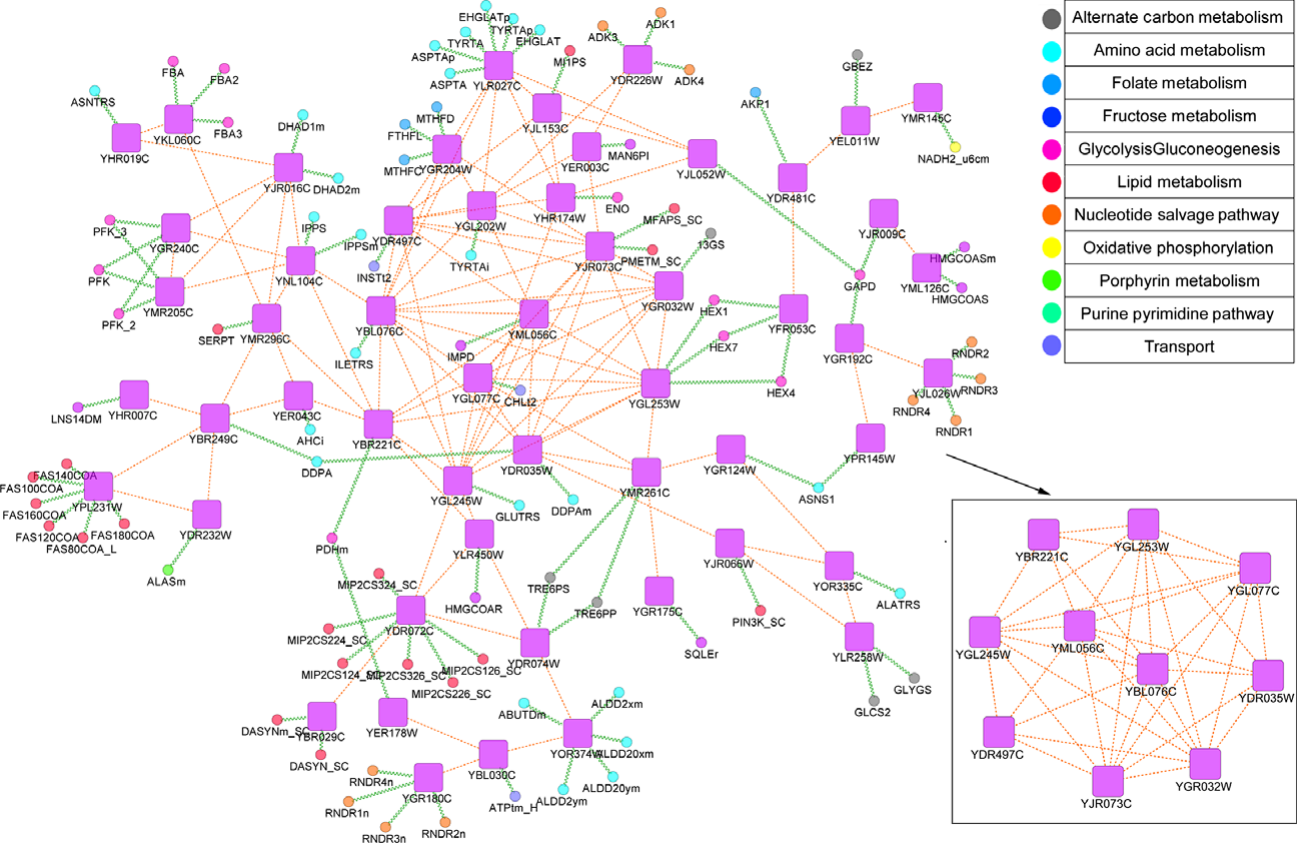
Coexpression network of metabolic genes based on the intra‐ and interspecies variation in gene expression. Only the main island of the coexpression network is shown. gra: gene‐reaction association; pp: coexpression relationships between metabolic genes.

We also wondered whether the metabolic genes in the model displayed the same patterns in expression profiles with each other. As a result, we built up a coexpression network based on the intra‐ and interspecies expression variation (Fig. 4; Methods). It shows that most of the coexpressed genes are associated with amino acid and lipid metabolism. Besides, 10 metabolic genes present a densely connected module, mainly participating in Glycolysis/Gluconeogenesis and Phospholipid Biosynthesis.

### Simulating phenotypic characteristics of metabolic model

Using the metabolic model, we could predict phenotypes of yeast metabolic genes. In the previous studies, one of the most important phenotypic predictions is the simulation of cellular growth 35. As a result, we defined biomass composition of the cell as the biomass objective function and performed FBA on the reconstructed network to maximize the objective function. It shows that the optimal solution for the model is the same as the optimal solution in *S. cerevisiae* metabolic network (optimal flux = 0.097).

We performed a genome‐scale single gene deletion study using both FBA and linearMOMA methods in COBRA toolbox 35. It shows that 113 genes were considered lethal and 491 genes were nonlethal out of 604 genes in the model, with 95 gene deletions leading to reduced maximal growth rates and 396 exhibiting no change in growth using FBA (Fig. S4A). The growth ratios of 331 gene deletions to wild‐type are different between two methods. It is mainly due to the different flux‐based analysis strategies by FBA and lMOMA 33. Compared with FBA, lMOMA is based on the same stoichiometric constraints, but the optimal growth flux for mutants is relaxed, providing an approximate solution for a suboptimal growth flux state. However, it shows that the lethal genes predicted using FBA are also predicted lethal using lMOMA, suggesting that prediction of essential metabolic genes from both methods are consistent. In addition, the single gene deletion results show that biomass could be produced in 451 out of 492 the gene‐deletion strains in which genes annotated as inessential or nonauxotrophic were deleted (true positive) and that biomass could not be produced in 48 mutant strains where these inessential or nonauxotrophic genes were deleted (false negative). As a result, the consensus metabolic model has a 90.4% sensitivity for identifying the essential genes. If the simulation results predicted that biomass could be produced following a gene deletion, the deleted gene was not considered as essential in 92% of the cases (a 92% positive predictive value).

We wondered the effects of reducing flux through reactions catalyzed by the highly expressed genes in consensus metabolic model. As a result, we studied the effect of decreasing the expression level of the top two genes (Fig. S4B) on the growth rate to predict haploinsufficient phenotypes. The gene YLR044C (pyruvate decarboxylase; EC 4.1.1) participates in Pyruvate Metabolism while the gene YKL060C (fructose‐bisphosphate aldolase; EC 4.1.2.13) catalyzed Glycolysis/Gluconeogenesis. The growth rate is sustained near the optimal value over a range of fluxes for Pyruvate Metabolism (PYRDC, Pyruvate decarboxylase) while the growth rate for Glycolysis/Gluconeogenesis (fructose‐bisphosphate aldolase) is sharply reduced after optimal value, indicating network robustness with respect to flux changes in PYRDC (Fig. S4B). However, a complete deletion of the PYRDC reaction would lead to a lethal phenotype, whereas deletion of the fructose‐bisphosphate aldolase reaction exhibits no change in growth.

### Applications of consensus model in metabolic engineering

One important use of metabolic reconstructions is to guide of metabolic engineering in yeast 42, for example, overproduction of biofuels 43. Here, we applied the model to identify how to increase *in silico* production of desired metabolites. We took yeast‐specific reactions and ethanol as examples.

We searched the Yeast Metabolome Database (http://www.ymdb.ca/) using metabolic genes in the metabolic model and identified 54 yeast‐specific reactions (Table S1). We set the minimal medium composition of the metabolic model aerobic and contain a glucose supply (20 mmol gDW^−1^ h^−1^) and predicted a list of candidate reactions for deletion to optimize product formation of these yeast‐specific reactions (Table 1). It shows that seven out of the 54 reactions can be optimized by reaction knockouts. Besides, it also shows that 2‐oxoglutarate exchange (EX_akg) can be knocked out for the overproduction of five specific reactions.

**Table 1:** Metabolic engineering of yeast‐specific reactions using *in silico* GDLS optimizations

	MFAPS	PETOHM	PINOS	PMETM	PSERDv	DAGPYP	PStv
Knockouts	EX_akg	EX_akg	EX_akg, HMGCOAtm	EX_akg	PStm	EX_akg, HMGCOAtm	PStm
Product	0.012	0.012	0.01	0.012	0.02	0.013	2.0 × 10^−4^
Biomass	1.95	1.95	1.95	1.95	1.95	1.95	1.95

The concentration of product and biomass is mmol gDW^−1^ h^−1^. EX_akg: 2 Oxoglutarate exchange; HMGCOAtm: Hydroxymethylglutaryl CoA reversible mitochondrial transport; PStm: phosphatidylserine mitochondrial transport; MFAPS: methylene fatty acyl phospholipid synthase; PETOHM: phosphatidylethanolamine N methyltransferase; PINOS: phosphatidylinositol synthase; PMETM: Phosphatidyl N methylethanolamine N methyltransferase; PSERDv: phosphatidylserine decarboxylase; DAGPYP: diacylglycerol pyrophosphate phosphatase; PStv: phosphatidylserine vacuolar transport.

We estimated the *in silico* overproduction of ethanol, zymosterol, and D‐sorbitol as biofuel production. The resulting knockout list for ethanol is aldehyde dehydrogenase acetaldehyde NADP, catalase, CO2 transport diffusion mitochondrial, glucose 6 phosphate isomerase, and threonine aldolase (Table 2). The resulting knockout predicted a growth rate of ~ 0.21 and a product excretion rate of ~ 37.29. As for zymosterol, the resulting knockout predicted a growth rate of ~ 0.44 while a product excretion rate of ~ 1.50. We set the maximum number of knockouts to be no limit, the list of reactions to knock out increases to 12 (the list for zymosterol in Table 2 plus ALATA_L, BPNT, GHMT2r, GLYt2 m, HSK, IPPS, NDPK1). However, the growth rate is reduced ~ 0.28 and the production rate increases to ~ 1.67. For D‐Sorbitol, The optimal knockouts are transketolase, L‐alanine transaminase, 3‐5‐bisphosphate nucleotidase, ergosterol exchange, and h2o transport via diffusion. Both ethanol and D‐Sorbitol result in the some similar optimal flux distribution.

**Table 2:** Metabolic engineering of consensus metabolic model for biofuel production

	Ethanol	Zymosterol	D‐Sorbitol
Knockout list	ALDD2y, CAT, CO2tm, PGI, THRA	CSNAT, ERGSTt, ME1 m, PYRt2 m, TKT2	ALATA_L, BPNT, EX_ergst(e), H2Ot, TKT2
Product	37.29	1.50	14.2
Biomass	0.21	0.44	0.12

The concentration of product and biomass is mmol gDW^−1^ h^−1^. ALDD2y: aldehyde dehydrogenase acetaldehyde NADP; CAT: catalase; CO2tm: CO2 transport diffusion mitochondrial; PGI: glucose 6 phosphate isomerase; THRA: Threonine aldolase; CSNAT: carnitine O acetyltransferase; ERGSTt: ergosterol reversible transport; ME1 m: malic enzyme NAD mitochondrial; PYRt2 m: pyruvate mitochondrial transport via proton symport; TKT2: transketolase; ALATA_L: L‐alanine transaminase; BPNT: 3‐5‐bisphosphate nucleotidase; EX_ergst(e): Ergosterol exchange; H2Ot: H2O transport via diffusion.

## Discussion

In the recent 10 years, network reconstruction approaches have developed rapidly 7, 44. The yeast metabolic reconstruction presented here represents an analogous process for systems biology studies of a target organism. With the successful achievement of the consensus reconstruction based on RNA‐seq, similar strategies should benefit systems biology for other organisms in metabolic modeling. We believe that the metabolic model reconstruction provided here will have special utility in a number of areas. First, the reconstruction will allow successful phenotype predictions, including cell growth, in response to genetic and/or environmental perturbations using a variety of methods 6. In the study, we simulated gene deletion phenotypes and robustness analysis of specific reactions. The results are of importance to studying molecular functions of metabolic genes. Second, we can perform an exploration of metabolic pathways and well‐curated connections between gene products. With the inevitable depletion of the world's energy supply, there has been an urgent need in alternative sources of energy. In the recent years, many scientists are increasingly conscious of biomass energy as a means of providing modern energy 45. The results show that we can apply the consensus metabolic model in biofuel production. Moreover, we can optimize the overproduction of specific metabolites (such as ethanol) through gene knockouts. Third, it can be integrated with other high‐throughput data, such as microarray 46, genomic information 47, and proteomics 48, for exploring questions related to comparative metabolomics and of metabolic pathway evolution.

Yeasts can cause a spectrum of diseases that range from colonization to uniformly fatal invasive disease. For example, Invasive fungal diseases (IFDs) are increasingly common complications in critically ill patients worldwide and are frequently fatal 49. Referring to yeast biology, several groups have stressed the threat that the lack of new antifungal drugs of broad spectrum and low toxicity poses to public health 50. We reason that the yeast metabolic model is suitable for drug development. There are several available strategies for this. First, the structures of 100 metabolic proteins have been solved to date, which can be easily applied in the drug design 51. Second, the systems biology tools, especially the constraint‐based modeling of genome‐scale metabolic networks, can be used in exploring pathogenic processes and drug discovery 52. In addition, we explored the conservation and divergence of the metabolic genes in gene expression. The majority of the consensus metabolic genes is conserved, which can provide useful evolutionary information for targets of broad‐spectrum therapeutics.

Despite our experimental and statistical rigor, our dataset does have some limitations. We used two RNA‐seq datasets with 20 samples to reconstruct the coexpression network, which may not cover the entire temporal differentially expressed genes. Besides, the reconstructed metabolic model is not robust to the changing flux through a single reaction. Some of the links between reactions or nodes (including metabolites, reactions, or genes) might be missing. The future work should focus on the filling gaps in metabolic pathways.

## Conclusion

In this study, we reconstructed a consensus metabolic model in four yeast species based on RNA‐Seq datasets. The metabolic model can be applied to metabolic engineering and benefit communities studying genome‐scale metabolic networks of other organisms.

## Author contributions

YZ, YW carried out the RNA‐seq data analysis, network simulations, and drafted the manuscript. LZ performed all the statistical analysis, and helped draft the paper. JH conceived of the study, and helped to draft the manuscript. All authors read and approved the final manuscript.

## Supporting information


**Fig. S1**. The phastCons tree model for four yeast species.
**Fig. S2.** The Gene Ontology molecular functions enrichment of transcripts *Saccharomyces cerevisiae* not included in the consensus dataset.
**Fig. S3.** The compartmentalization of the consensus metabolic model.
**Fig. S4**. Network simulation of phenotypic characteristics.
**Table S1**. Yeast‐specific reactions in consensus metabolic model.

**Text S1.** The consensus metabolic model for four yeast species in Systems Biology Markup Language.
