# Supplementary material for: Reconstruction and applications of consensus yeast metabolic network based on RNA sequencing
Source: FEBS Open Bio. 2016 Feb 27;6(4):264–75. doi: 10.1002/2211-5463.12033 (PMC4821349; doi:10.1002/2211-5463.12033)
Supplement: Supplementary file 1 — Fig. S1. The phastCons tree model for four yeast species. Fig. S2. The Gene Ontology molecular functions enrichment of transcripts Saccharomyces cerevisiae not included in the consensus dataset. Fig. S3. The compartmentalization of the consensus metabolic model. Fig. S4. Network simulation of phenotypic characteristics. Table S1. Yeast‐specific reactions in consensus metabolic model. [file FEB4-6-264-s001.pdf]

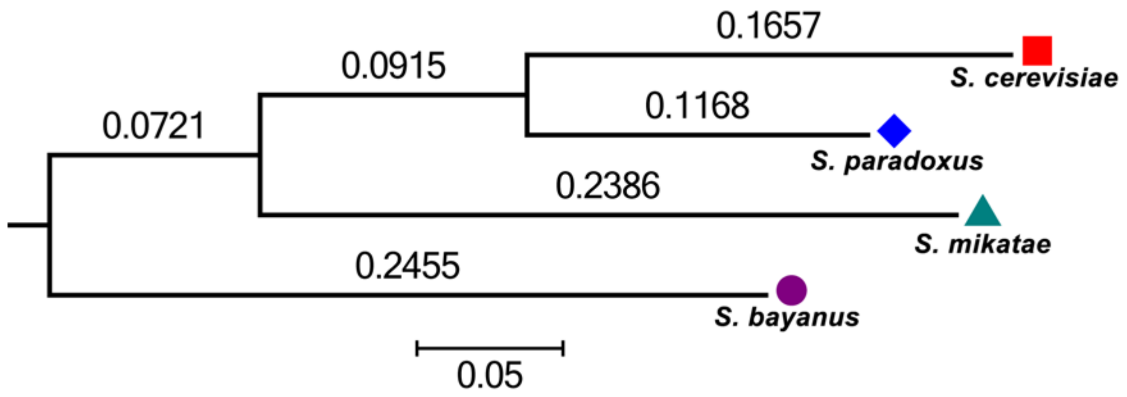

Figure S1. The phastCons tree model for four yeast species.

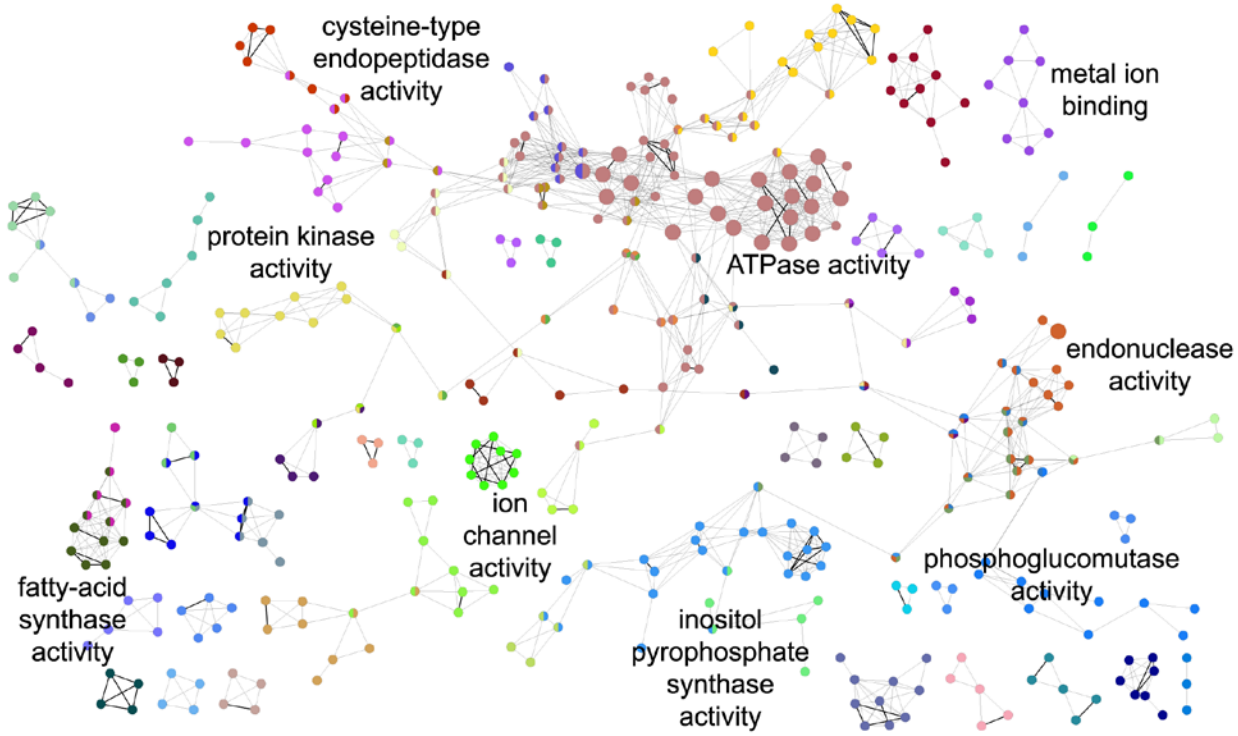

Figure S2. The Gene Ontology molecular functions enrichment of transcripts *S. cerevisiae* not included in the consensus dataset. The enrichment analysis was performed using ClueGO. The different colors correspond to different terms, with Fisher's exact test P<0.01.

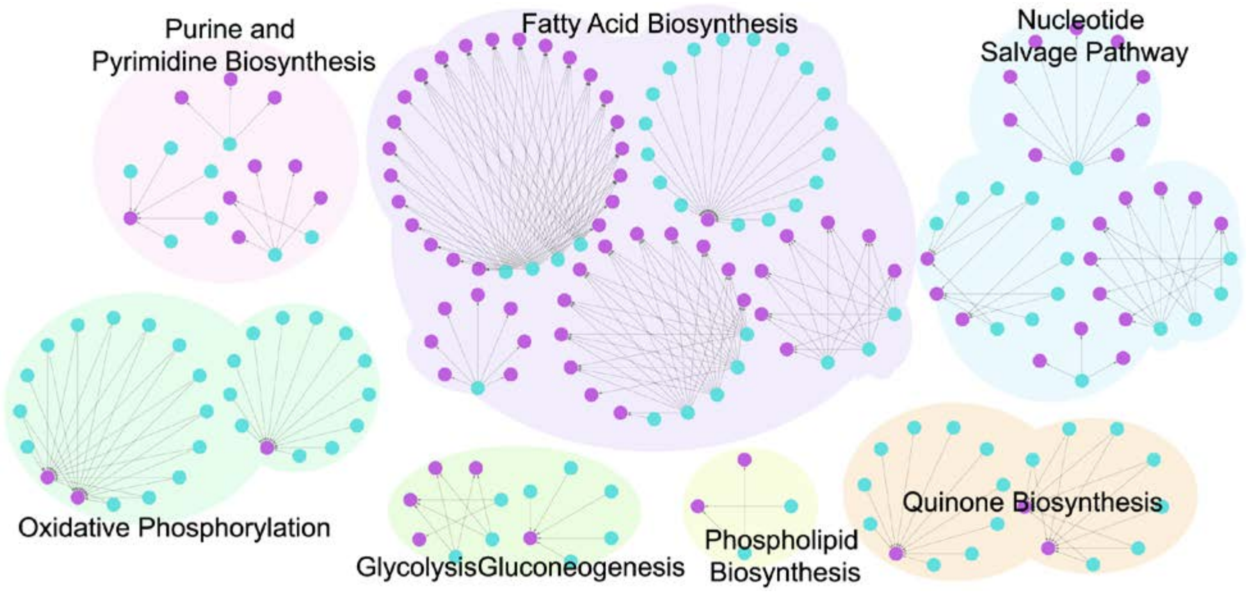

Figure S3. The compartmentalization of the consensus metabolic model. The green nodes represent metabolic genes while the purple ones are reactions.

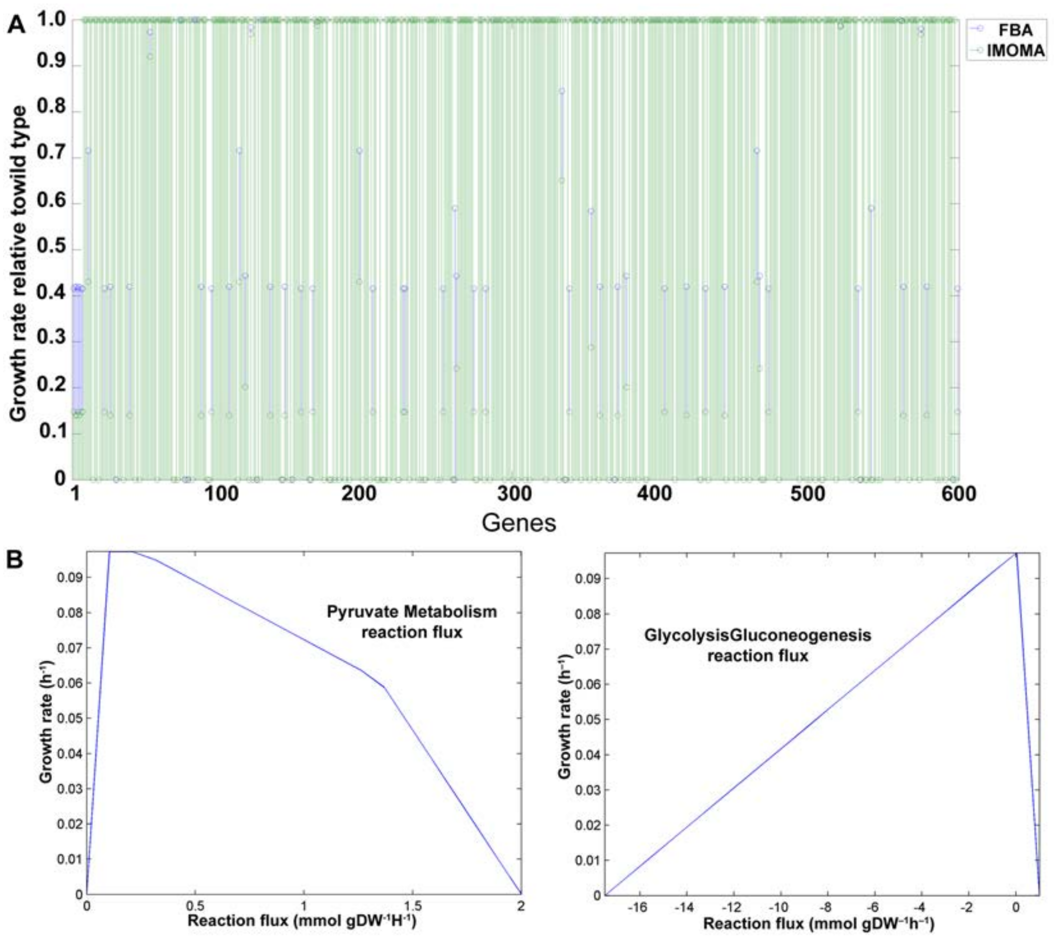

Figure S4. Network simulation of phenotypic characteristics. (A) Simulating single gene deletion phenotypes. The x axis represents metabolic genes in consensus metabolic model while the y axis represents growth rate of gene deletions relative to wild type. **(B)** The sensitivity of the predicted growth rate to changing the flux through Pyruvate Metabolism (left) and GlycolysisGluconeogenesis (right).

Table S1. Yeast-specific reactions in consensus metabolic model.

Rxn description	Genes	Subsystem
acyldihydroxyacetonephosphate reductase yeast specific		Phospholipid Biosynthesis
1 Acyl glycerol 3 phosphate acyltransferase yeast specific	YDL052C	Phospholipid Biosynthesis
CDP diacylglycerol serine O phosphatidyltransferae yeast specific mitochondrial	YCL004W	Phospholipid Biosynthesis
diacylglycerol cholinephosphotransferase yeast specific	YHR123W YNL130C	Phospholipid Biosynthesis
diacylglycerol pyrophosphate phosphatase yeast specific	YDR284C YDR503C	Phospholipid Biosynthesis
CDP Diacylglycerol synthetase yeast specific	YBR029C	Phospholipid Biosynthesis
CDP Diacylglycerol synthetase yeast specific mitochondrial	YBR029C	Phospholipid Biosynthesis
Ethanolaminephosphotransferase yeast specific	YHR123W	Phospholipid Biosynthesis
glycerol 3 phosphate acyltransferase glycerol 3 phosphate yeast specific		Phospholipid Biosynthesis
glycerol 3 phosphate acyltransferase glycerone phosphate yeast specific		Phospholipid Biosynthesis
Inositol phosphorylceramide synthase ceramide 1 24C yeast specific	YKL004W	Sphingolipid Metabolism
Inositol phosphorylceramide synthase ceramide 1 26C yeast specific	YKL004W	Sphingolipid Metabolism
Inositol phosphorylceramide synthase ceramide 2 24C yeast specific	YKL004W	Sphingolipid Metabolism
Inositol phosphoryceramide synthase ceramide 2 26C yeast specific	YKL004W	Sphingolipid Metabolism
Inositol phosphorylceramide synthase ceramide 3 24C yeast specific	YKL004W	Sphingolipid Metabolism
Inositol phosphorylceramide synthase ceramide 3 26C yeast specific	YKL004W	Sphingolipid Metabolism
lipid phosphate phosphatase yeast specific	YDR284C YDR503C	Phospholipid Biosynthesis
methylene fatty acyl phospholipid synthase yeast specific	YJR073C	Phospholipid Biosynthesis
mannose inositol P 2 ceramide synthase ceramide 1 24C yeast specific	YDR072C	Sphingolipid Metabolism
mannose inositol P 2 ceramide synthase ceramide 1 26C yeast specific	YDR072C	Sphingolipid Metabolism
mannose inositol P 2 ceramide synthase ceramide 2 26C yeast specific	YDR072C	Sphingolipid Metabolism
mannose inositol P 2 ceramide synthase ceramide 2 24C yeast specific	YDR072C	Sphingolipid Metabolism
mannose inositol P 2 ceramide synthase ceramide 3 24C yeast specific	YDR072C	Sphingolipid Metabolism
mannose inositol P 2 ceramide synthase ceramide 3 26C yeast specific	YDR072C	Sphingolipid Metabolism
mannose inositiol phophorylceramide synthase ceramide 1 24C yeast specific	YBR036C YPL057C	Sphingolipid Metabolism
mannose inositol phosphorylceramide synthase ceramide 1 26C yeast specific	YBR036C YPL057C	Sphingolipid Metabolism
mannose inositol phosphorylceramide synthase ceramide 2 24C yeast specific	YBR036C YPL057C	Sphingolipid Metabolism
mannose inositol phosphorylceramide synthase ceramide 2 26C yeast specific	YBR036C YPL057C	Sphingolipid Metabolism
mannose inositol phosphorylceramide synthase ceramide 3 24C yeast specific	YBR036C YPL057C	Sphingolipid Metabolism
mannose inositol phosphorylceramide synthase ceramide 3 26C yeast specific	YBR036C YPL057C	Sphingolipid Metabolism
phosphatidate kinase yeast specific		Phospholipid Biosynthesis
phosphatidate reversible transport mitochondrial yeast specific		Transport Mitochondrial
phosphatidylethanolamine N methyltransferase yeast specific	YGR157W	Phospholipid Biosynthesis
phosphatidylethanolamine Golgi transport yeast specific		Transport Golgi Apparatus
phosphatidylethanolamine mitochondrial transport yeast specific		Transport Mitochondrial
phosphatidylethanolamine vacuolar transport yeast specific		Transport Vacuolar
phosphatidylglycerol phosphate phosphatase A yeast specific mitochondrial		Phospholipid Biosynthesis

phosphatidylinositol 3 phosphate 4 kinase yeast specific		Phospholipid Biosynthesis
1 phosphatidylinositol 4 5 bisphosphate phosphodiesterase yeast specific	YPL268W	Phospholipid Biosynthesis
1 phosphatidylinositol 3 kinase yeast specific	YJR066W YKL203C YLR240W	Phospholipid Biosynthesis
phosphatidylinositol 4 kinase yeast specific	YLR305C YNL267W	Phospholipid Biosynthesis
phosphatidylinositol synthase yeast specific	YPR113W	Phospholipid Biosynthesis
Phosphatidyl N methylethanolamine N methyltransferase yeast specific	YJR073C	Phospholipid Biosynthesis
phosphatidylserine decarboxylase yeast specific Golgi	YGR170W	Phospholipid Biosynthesis
phosphatidylserine decarboxylase yeast specific mitochondrial	YNL169C	Phospholipid Biosynthesis
phosphatidylserine decarboxylase yeast specific vacuolar	YGR170W	Phospholipid Biosynthesis
phosphatidylserine synthase yeast specific	YER026C	Phospholipid Biosynthesis
phosphatidylserine synthase yeast specific mitochondrial	YER026C	Phospholipid Biosynthesis
phosphatidylserine Golgi transport yeast specific		Transport Golgi Apparatus
phosphatidylserine mitochondrial transport yeast specific		Transport Mitochondrial
phosphatidylserine vacuolar transport yeast specific		Transport Vacuolar
phosphatidyl 1D myo insoitol nuclear transport yeast specific		Transport Nuclear
phosphatidyl 1D myo 4 inositol nuclear transport yeast specific		Transport Nuclear
thiazole phosphate synthesis xylulose 5 phosphate yeast specific		Thiamine Metabolism
